# Epidemiology and Impact of Anti-Pneumococcal Vaccination and COVID-19 on Resistance of *Streptococcus pneumoniae* Causing Invasive Disease in Piedmont, Italy

**DOI:** 10.3390/antibiotics13080740

**Published:** 2024-08-06

**Authors:** Alessandro Bondi, Emanuele Koumantakis, Antonio Curtoni, Anna Maria Barbui, Marco Peradotto, Daniela Lombardi, Roberto Casale, Silvia Alizzi, Elisa Zanotto, Lorena Charrier, Rossana Cavallo, Cristina Costa

**Affiliations:** 1 Microbiology and Virology Unit, AOU Città della Salute e della Scienza di Torino, 10126 Turin, Italy; alessandro.bondi@unito.it (A.B.); antonio.curtoni@unito.it (A.C.); annainbici@libero.it (A.M.B.); mperadotto@cittadellasalute.to.it (M.P.); salizzi@cittadellasalute.to.it (S.A.); rossana.cavallo@unito.it (R.C.); 2 Department of Public Health and Pediatrics, University of Turin, 10126 Turin, Italy; emanuele.koumantakis@unito.it (E.K.); roberto.casale@unito.it (R.C.); lorena.charrier@unito.it (L.C.); 3 Post Graduate School of Medical Statistics, University of Turin, 10126 Turin, Italy; 4 Regional Epidemiology Reference Service for the Surveillance, Prevention and Control of Infectious Disease (SeREMI), 15121 Alessandria, Italy; dlombardi@aslal.it

**Keywords:** *S. pneumoniae*, anti-pneumococcal vaccination, antimicrobial resistance

## Abstract

Background: The international surveillance of antimicrobial resistance (AMR) reports *S. pneumoniae* as one of leading causes of death associated with AMR. Against invasive disease, several vaccinations are available and a reduction in AMR in *S. pneumoniae* has been observed. Here, we evaluated the impact of anti-pneumococcal vaccination policy and the SARS-CoV2 outbreak on AMR in *S. pneumoniae* causing invasive disease. Methods: We collected all strains of *S. pneumoniae* causing invasive disease from 2008 in the Piedmont region (Italy). Each strain was typed in order to identify the serogroup and data about AMR were collected. The population under surveillance was classified as infants, children, adults, and the old population. Results: We collected *n* = 2076 *S. pneumoniae* strains, with 21.9% and 40.3% being resistant to penicillin G and erythromycin, respectively. We reported an increased risk of infection with penicillin-resistant *S. pneumoniae* among all populations and evaluated whether the infection was caused by a serotype included in the vaccine formulation. A similar increase was observed after the SARS-CoV2 outbreak. Conclusions: In the Piedmont region, subsequently to the introduction of anti-pneumococcal vaccination, a significant increase in the risk of penicillin G-resistant invasive pneumococcal disease among infants and old population was reported. No significant impact was found for the SARS-CoV2 outbreak.

## 1. Introduction

*Streptococcus pneumoniae* (pneumococcus) is a Gram-positive diplococcus that colonizes the upper respiratory tract [1]. It is the aetiological agent of several acute infections, like sinusitis, otitis, community-acquired pneumonia (CAP), and life-threatening infections, e.g., meningitis and bacteremic pneumonia, known as invasive pneumococcal infection (IPD) [2]. The burden of disease is higher among the elderly (over 65 years), young children (under 5 years), and all patients with an immunocompromised condition and comorbidities [3]. It has been reported that 30% of all CAPs are caused by *S. pneumoniae*, with a burden of a high mortality rate (11–40%) [4]. Several anti-pneumococcal vaccines have been available since 1983, such as the 23-valent pneumococcal polysaccharide vaccine (PPV-23) and different formulations of the pneumococcal conjugate vaccine (PCV-7, PCV-10, and PCV-13) [5]. Vaccination reduced the cases of IPD by 64% among children younger than 5 years, and a herd effect was observed, with a reduction in invasive disease among the adult population [6]. Despite this, IPDs are still widespread in the population, mainly caused by non-vaccine serotypes [7]. Beta-lactam antibiotics (e.g., penicillin and ceftriaxone) are the first-line treatment for invasive disease. In cases of resistant *S. pneumoniae* strains or intolerance to beta-lactams, macrolides, fluoroquinolones, and vancomycin can be administered [8]. The rise in antimicrobial resistance is a major global concern, and *S. pneumoniae* is the fourth leading bacterial pathogen causing antimicrobial resistance (AMR)-associated death, with more than 600,000 cases reported in 2019 [9]. A recent review reported that 34%, 53%, and 14% of isolated *S. pneumoniae* are non-susceptible to penicillin (PG), macrolides (MCs), and third-generation cephalosporins (3GCs), respectively, in the paediatric population [10]. A high antibiotic resistance level has also been reported in the adult population, both in Europe and in the United States, especially for PG and MCs [11,12]. The highly inappropriate prescription of antibiotics is considered one of the causes of increased AMR in *S. pneumoniae* [12]. Despite this, it appears that anti-pneumococcal vaccination may play a role in this trend. Some authors suggest that low vaccination rates could explain the level of AMR in the adult population [12,13,14]. Following the introduction of PCV, a reduction in penicillin-non-susceptible strains has been observed in several settings, suggesting protection against IPD caused by *S. pneumoniae* AMR [15,16,17]. However, a subsequent expansion of non-PCV strains causing IPD has been observed simultaneously with the appearance of resistant strains, especially for certain serotypes such as 11A [18,19,20]. Another cause of the development of AMR in *S. pneumoniae* could be the SARS-CoV-2 pandemic. The increased use of antibiotics to avoid bacterial co-infection could be related to the development of AMR in several bacterial species, including *S. pneumoniae* [21,22]. In Italy, recent data are available on AMR surveillance in *S. pneumoniae* [23,24,25,26], and vaccination is based on local policy decisions. Notably, in the Piedmont region, PVC-13 vaccination has been mandatory for newborns since 2010, and PPV-23 has been available for the population over 65 years of age since 2017. No data are available for our region on the impact of vaccination and AMR in *S. pneumoniae.* On the other hand, the impact of SARS-CoV-2 on IPD has been previously evaluated, without highlighting its impact on AMR [27]. Hence, this study aims to describe the epidemiology of the antibiotic resistance of *S. pneumoniae* strains causing IPD in Piedmont, Italy. Specifically, the first objective is to evaluate the impact of anti-pneumococcal vaccination. As a secondary objective, we considered the contribution of the SARS-CoV-2 pandemic to the spread of non-susceptible strains of *S. pneumoniae*.

## 2. Results

Over the period between 2008 and 2022, 2076 strains of *S. pneumoniae* causing IPD were collected in the Piedmont region and referred to the Microbiology and Virology Unit of the University Hospital Città della Salute e della Scienza di Torino. Sample features are described in Table S1, available in the Supplementary Material. Most were isolated from elderly patients (*n* = 1334, 64.3%), followed by adults (*n* = 550, 26.5%), children (*n* = 118, 5.7%), and infants (*n* = 74, 3.6%) (Table 1). Approximately 13% of IPDs were meningitis (*n* = 264), while sepsis was present in 44% of all cases (*n* = 913). The development of sepsis was more frequent in infants and children than in adults and the old population. Except for third-generation cephalosporins and levofloxacin, which showed a low grade of antimicrobial resistance, we observed a reduced level of susceptibility to penicillin and erythromycin in all age groups. In particular, a moderate degree of resistance to PG was observed in newborns (21.9%). Erythromycin showed a reduced level of susceptibility in all age groups, particularly in infants (40.3%). Resistance to at least two classes of antibiotics was more common in infants compared to the other age groups.

**Figure 1:**
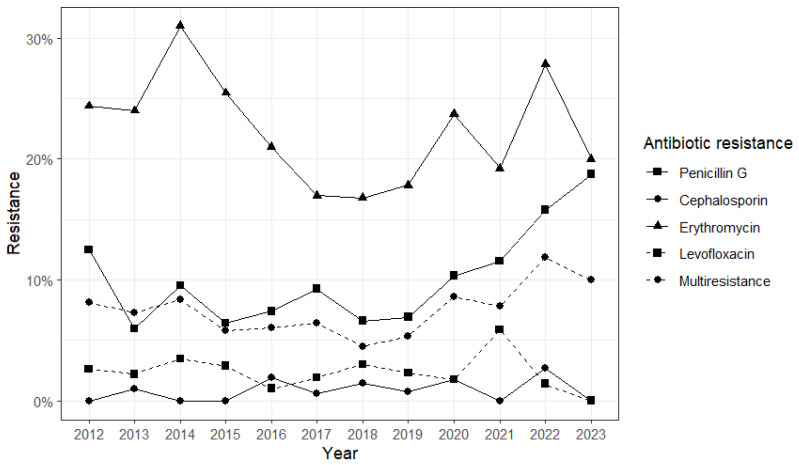
Prevalence of antibiotic resistance in the elderly group in the period 2012–2023. Different point shapes and line types represent data related to resistance to each evaluated antibiotic and multiresistance.

The distribution of AMR by year is shown in Figure S1. In brief, we noted a reduction in PG-R in the infant group during the surveillance period. Penicillin G (PG) resistance was reported from 2010 to 2017, with no cases between 2018 and 2023, except for 2022 (*n* = 2, 40%). No cases of third-generation cephalosporin (3GC) resistance and Levofloxacin (LVX) resistance were reported from 2008 to 2023. Resistance to erythromycin (E) was widely reported until 2017, while only one case was registered in both 2021 (100%) and 2022 (20%). Among children, the majority of PG-R was reported from 2021 to 2023 (*n* = 5, 56%). No 3GC-R strain was reported during the surveillance period. The same was observed for LVX-R, except for 2010 (*n* = 1, 8%). The adult group showed an increase in PG-R from 2015 (*n* = 1, 2%) to 2023 (*n* = 3, 17%), as well as a very low diffusion of 3GC-R and LVX-R. Resistance to erythromycin was widespread among *S. pneumoniae* during the surveillance period. Finally, in the elderly, an increase in penicillin G resistance was found: from 12.5% in 2012 to 18.8% in 2023 (Figure 1). A more limited increase was observed for multiresistance, from 8.1% in 2012 to 10% in 2023. On the other side, resistance to cephalosporin and erythromycin and multiresistance seemed stable.

Children had the highest proportion of IPD cases caused by the PCV13 strain (59.3%), followed by newborns (44.6%), the elderly population (35.6%) and adults (33.1%). The three most common serotypes were 8 (*n* = 314, 15.1%), 3 (*n* = 298, 14.4%), and 14 (*n* = 98, 4.7%). All serotypes are reported in Supplemental Materials (Table S2). Serotype 3 was the most prevalent in the elderly population (16.4%), 8 in newborns and adults (13.5% and 17.8% respectively), and 14 in children (7.6%). The overall resistance for the four antimicrobials is reported in Table 2. We found a high level of erythromycin resistance (21.2%) and PG resistance (8.8%), while a lower rate was detected for 3GC and LVX. When considering specific serotypes, a reduced susceptibility was reported for PG and E in serotype 14 (36.8% and 72.6% respectively) with a high diffusion of the MR phenotype compared to all serotypes (31.6% vs. 6.3%). Serotype 8 showed low levels of antibiotic resistance for all classes compared to the overall serotypes.

A significant increase in the risk of PG-resistant *S. pneumoniae* infection was observed among infants and children after the 2010 vaccination policy (OR: 3.87; 95%CI: 1.29–12.8; *p* = 0.019), and the same increase, although not significant, was observed for the risk of multiresistance (OR: 3.78; 95%CI: 1.00–16.9; *p* = 0.060). In all analyses, infants were found to be at higher risk for antibiotic-resistant IPD than children; moreover, being infected by the same serotype included in the PCV13 vaccine emerged as a risk factor for antibiotic resistance (Table 3).

**Figure 2:**
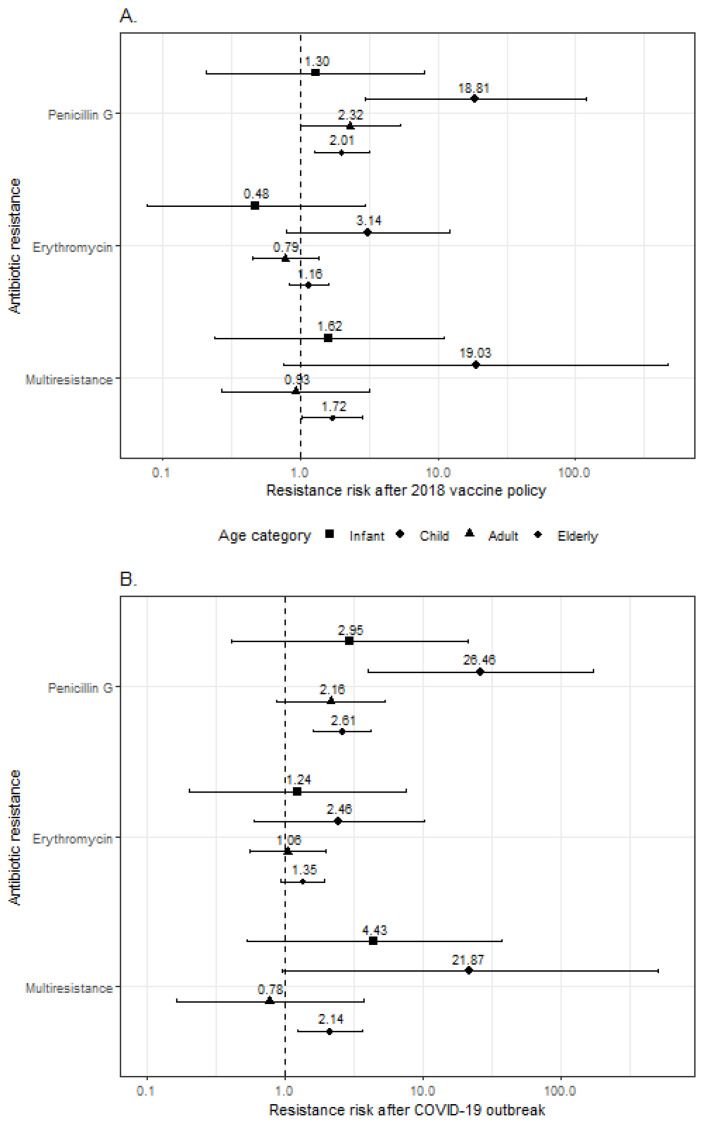
Forest plot showing the results from logistic regressions evaluating the association between 2018 vaccine policy (**A**) and COVID-19 pandemic (**B**) and antimicrobial resistance (to penicillin G, to erythromycin, and multiresistance). Odds ratios (dots) and 95% confidence intervals (horizontal bars) are provided with different symbols for each age group.

After the 2017 vaccination policy, the elderly showed an increased risk of penicillin G-resistant (OR: 2.01; 95%CI: 1.27–3.18; *p* = 0.003) and multiresistant (OR: 1.72; 95%CI: 1.02–2.86; and *p* = 0.038) infection compared with the pre-policy period (Figure 2A). Adults and children were at higher risk of PG-resistant IPD (respectively, OR: 2.32, 95%CI: 1.01–5.41, *p* = 0.047, and OR: 18.8, 95%CI: 3.27–156, and *p* = 0.002). A non-significant risk increase emerged among children in relation to erythromycin resistance (OR: 3.14; 95%CI: 0.77–12.5; and *p* = 0.102) and multiresistance (OR: 19.0; 95%CI: 0.77–473; and *p* = 0.072). No significant association between vaccination policy and resistance to the considered antibiotics was found among infants.

Similar results emerged when antibiotic resistance in patients with IPD after the COVID-19 pandemic outbreak was compared to that before the pandemic (Figure 2B). The elderly showed an increased risk of PG-resistant (OR: 2.61; 95%CI: 1.60–4.25; *p* < 0.001) and multiresistant IPD (OR: 2.14; 95%CI: 1.24–3.69; *p* = 0.007). Adults showed a non-significant association between the pandemic and penicillin G-resistant infection (OR: 2.16; 95%CI: 0.87–5.32; *p* = 0.096), which, conversely, was statistically significant in children (OR: 26.5; 95%CI: 4.05–173; and *p* = 0.001). The COVID-19 pandemic outbreak was not significantly associated with erythromycin resistance in any of the age groups.

## 3. Discussion

Despite the spread of vaccination, *Streptococcus pneumoniae* remains a major cause of IPD in the paediatric population and in the community. The invasive infection can be treated with several classes of antibiotics, such as penicillin, cephalosporins, macrolides, and fluoroquinolones; however, the spread of antimicrobial resistance is described as a major global concern [9,10]. The surveillance of IPD is essential in order to evaluate vaccine efficacy, monitor the diffusion of resistant strains, and identify emerging serotypes. To achieve these goals, active surveillance of pediatric IPD has been ongoing in the Piedmont region since 2008. Moreover, from 2012 the surveillance has been extended to the whole population. However, data on the prevalence of AMR and the impact of PCV vaccination in Piedmont have not yet been published. In the present study, we evaluated the distribution of AMR among *S. pneumoniae* isolated from invasive infections and the impact of PCV vaccination. Our study included 15 years of data for the paediatric subgroup and 11 for adults. We observed a high diffusion of PG non-wild-type strains among infants compared to other age groups. The overall rate of non-wt *S. pneumoniae* is 21.9%. Similar results were shown in an international survey, which reported a 20% reduced sensitivity to PG in high-income Western European countries [10], which was low compared with France and the US [11,28]. The heterogeneity in AMR distribution could be due to the dissemination of a different *S. pneumoniae* strain, but no data about serotyping are available. High levels of erythromycin resistance have been reported in several epidemiological surveys and the data are similar in Europe and the US [10,11,28]. One case of 3GC resistance has been reported over 15 years of surveillance. This finding is in agreement with data from the literature, which show a low prevalence of 3GC resistance [10,11,28]. Among adults, PG resistance was lower than among children and infants, consistent with evidence from the study conducted by Mohanty and colleagues [28,29]. IPD surveillance in France showed a similar spread of penicillin resistance among the over-18 and child populations [11]. Worldwide, a lower prevalence of erythromycin resistance has been reported in adults and the elderly than in children and infants [11,28,29], and this was confirmed by our results. The resistance to 3GC and LVX in the over-18 population is not widespread. Regarding the impact of PCV vaccination on AMR, a reduction in PG-R was observed, but several studies have reported an increase in AMR caused by serotype replacement after vaccine selective pressure [15,16,17,18,19,20]. Nevertheless, a recent systematic review found no difference in AMR patterns after PCV introduction [19].

We found an increased risk for the infant population to be infected with the *S. pneumoniae* PG-R strain after the introduction of the vaccination policy. On the other hand, we noted a reduction in the PG-R strain after 2018, with the exception of 2022. The increased risk could be explained by the small number of IPDs collected from 2008 to 2010 compared to the post-PCV introduction period. We found that PCV-Sp is associated with a higher risk of AMR compared to n-PCV-Sp, in line with the literature [19]. With regard to the adult and elderly subgroups, we observed an increase in the diffusion of PG and E resistance among those over 65 years old, associated with an increased risk of PG-R strain infection after the introduction of the PPV-23 vaccine. The same increase has been observed in the literature, with the occurrence of an increase in not-susceptible *S. pneumoniae* following the introduction of the PCV vaccine [19,29]. This suggests that the same effect could also be observed for the PPV-23 vaccine. It is known that the PCV vaccination of children can lead to a reduction in AMR incidence in the adult population [19]. The use of PPV-23 could change the serotype epidemiology, with different consequences across the geographic areas.

Regarding the impact of SARS-CoV2 on *S. pneumoniae* AMR, little data are available. Sempere and colleagues highlight an increase in Pen-R in 11A serotype [19]. In our study, we observed no significant impact on the increase in AMR; however other data are necessary to confirm this result. During the COVID-19 period, excessive and incorrect use of macrolides [i.e., azithromycin] occurred, and this could have led to a reduction in susceptibility among bacteria populations (i.e., *E. coli* and *N. gonorrhoeae*) [30]. The increase in AMR observed could be explained as a consequence of vaccination policy and not SARS-CoV2. However, our findings did not suggest an increased risk of E-R after the COVID-19 pandemic outbreak.

Furthermore, despite mixed results reported in the literature, our findings suggest an effect of vaccination and, potentially, the COVID-19 outbreak on *S. pneumoniae* serotype resistance to antibiotics in Piedmont. Regional surveillance of AMR and vaccine coverage should be considered in implementing the best immunization policy and vaccine formulation choice. In addition, to have more robust data for a better description of the impact of pneumococcal vaccination, multicenter studies involving regions or countries that share the same vaccination policies are desirable.

This study presents some limitations. Data collection and sample sizes among the four age groups under study were quite heterogeneous, potentially affecting the interpretation of our findings. To overcome this issue, we stratified regression analyses, when feasible. AMR could be influenced by other factors, i.e., antibiotic use, the presence of clonal bacterial population, and the general level of AMR diffusion before the surveillance period, that were not considered in this study. However, our study collected regional data over fifteen years, enabling a more precise investigation of the effect of vaccination policies and the outbreak of the COVID-19 pandemic on *S. pneumoniae* antibiotic resistance in the Piedmont region. Additionally, the same stratification based on age groups allowed us to observe and describe different facets of the phenomenon.

In conclusion, following the introduction of anti-pneumococcal PCV-13 vaccination, we reported a significant increased risk of penicillin G-resistant IPD among infants. The same increase was observed among the old population after PPV-23 introduction. In contrast, no significant risk was found for the other antibiotics. The SARS-CoV2 pandemic seems to have had no impact on the AMR of *S. pneumoniae* and the risk of IPD caused by a resistant strain.

## 4. Materials and Methods

### 4.1. Clinical Data and Strain Collection

IPD *S. pneumoniae* strains isolated in the Piedmont region were sent to the Microbiology Unit of Città della Salute e della Scienza University Hospital. The strain was accompanied by an IPD notification sheet. IPD is an infectious disease defined as the recovery of pneumococcus from sterile body sites: blood (B), pleural fluid (PF) and cerebrospinal fluid (CSF). Notification of paediatric IPD cases and subsequent serotyping of *S. pneumoniae* strains has been mandatory in the Piedmont region since 2008. Since 2012, surveillance has been extended to all age cases of IPD. The notification sheet contains the following information: patient age, clinical picture (bacteremia and/or meningitis with or without sepsis), and antimicrobial susceptibility to PG, 3GC, E, and LVX. Four age groups were considered: (i) infants (from 0 to 2 years old); (ii) children (>2 and ≤16 years old); (iii) adults (>16 and ≤64 years old); and (iv) the old population (over 64 years old).

### 4.2. S. pneumoniae Serotyping

All *S. pneumoniae* strains from 2008 causing IPD were collected at the Microbiology and Virology Unit of the University Hospital Città della Salute e della Scienza di Torino for serotype determination. Typing was performed by agglutination using specific antisera (rabbit anticapsular antibodies), following the manufacturer’s instruction (Pneumotest Kit, Serum State Institute, Copenhagen, Denmark). In brief, after overnight incubation on blood agar in an enriched CO_2_ atmosphere, one single colony was picked up and mixed with 2 µL of antisera. The agglutination (characterized by the formation of cells aggregates) was observed with the aid of optical microscopy. We classified the *S. pneumoniae* serotype into three classes: (i) PCV-Sp, to which belong the 1, 3, 4, 5, 6A, 6B, 7F, 9V, 14, 18C, 19A, 19F, and 23F serotypes; (ii) nPCV-Sp, which groups all nonPCV serotypes. In 2017, the anti-pneumococcal vaccination was introduced to those over 65 years old; it is voluntary based, with a PPV-23 formulation (which includes 1, 2, 3, 4, 5, 6A, 6B, 7F, 8, 9N, 9V, 10A, 11A, 12F, 14, 5B, 17F, 18C, 19A, 19F, 20B, 22F, 23F, and 33F serotypes).

### 4.3. Antimicrobial Resistance Data and Definitions

The antimicrobial resistance data were collected through notification of IPD cases, with reporting of both the minimum inhibitory concentration value (MIC) and the interpretation according to the European Committee for Antimicrobial Susceptibility Testing (EUCAST). To simplify the analysis, the three interpretative categories, resistant, intermediate, and susceptible, were grouped into (i) resistant (R) and (ii) susceptible (S). Specifically, for benzylpenicillin, the strain was defined as S if the MIC value was below the epidemiological cut-off values (0.06 mg/L (T) ECOFFs) [31]. This classification allowed us to separate wild-type from non-wild-type microorganisms. On the other hand, for third-generation cephalosporin, levofloxacin, and erythromycin, strains with MICs below the resistant breakpoint value (either intermediate or susceptible) were classified as S. If the antibiotic susceptibility profile showed resistance to ≥2 molecules, the strain was classified as a multiresistant (MR) strain.

### 4.4. Statistical Analysis

Medians and interquartile ranges (IQRs) for quantitative variables, and absolute and relative (%) frequencies for qualitative variables, were calculated. Differences among the four age groups were assessed using the Kruskal–Wallis test for continuous variables and the chi-square test for categorical variables. In addition, the reporting of antibiotic resistance variables was stratified according to the most frequent *S. pneumoniae* strains. Finally, for the elderly group, the number of different antibiotics subject to resistance was aggregated into annual percentages to describe potential fluctuations over time. To assess the association between vaccine policy implementation and antibiotic resistance, we fitted logistic regression models stratified by age group, where the dependent variable was resistance to antibiotics taken one at a time or multiresistance, and the independent variable was the vaccination policy of 2017. Before 2012, data on adults and the elderly were not available, so the association between the 2010 vaccine policy and antibiotic resistance was performed only for infants and children, setting the age group as a covariate. The significance level of 0.05 was set. Analyses were conducted using R software version 4.3.0 [28].

## Figures and Tables

**Table 1:** Sample characteristics stratified by age group. Data are absolute frequencies and percentages (%) if not otherwise stated. IQR: interquartile range; yo: years old.

	Infant (≤2 yo) (*n* = 74)	Child (>2 and ≤16 yo) (*n* = 118)	Adult (>16 and ≤64 yo) (*n* = 550)	Elderly (>64 yo) (*n* = 1334)	*p*-Value
Age (years), median [IQR]	0.644 [0.296–1.12]	5.00 [3.42–7.22]	52.1 [43.1–59.5]	77.0 [70.6–83.7]	<0.001
After 2010 vaccination policy	51 (68.9%)	67 (56.8%)	546 (99.3%)	1329 (99.6%)	<0.001
After 2018 vaccination policy	13 (17.6%)	15 (12.7%)	180 (32.7%)	431 (32.3%)	<0.001
After COVID-19 outbreak	10 (13.5%)	13 (11.0%)	104 (18.9%)	269 (20.2%)	0.058
Sepsis	52 (70.3%)	87 (73.7%)	242 (44.0%)	532 (39.9%)	<0.001
Meningitis	10 (13.5%)	20 (16.9%)	102 (18.5%)	132 (9.9%)	<0.001
Serotype included in the PCV13 vaccine	33 (44.6%)	70 (59.3%)	182 (33.1%)	475 (35.6%)	<0.001
Serotype included in the PCV10 vaccine	24 (32.4%)	54 (45.8%)	86 (15.6%)	190 (14.2%)	<0.001
Serotype					
3	3 (4.1%)	8 (6.8%)	69 (12.5%)	219 (16.4%)	<0.001
8	10 (13.5%)	7 (5.9%)	98 (17.8%)	199 (14.9%)	
12F	0 (0%)	10 (8.5%)	44 (8.0%)	95 (7.1%)	
Other	50 (67.6%)	86 (72.9%)	267 (48.5%)	668 (50.1%)	
Not typed	11 (14.9%)	7 (5.9%)	72 (13.1%)	153 (11.5%)	
Penicillin G resistance	14 (21.9%)	8 (8.6%)	29 (6.5%)	97 (8.9%)	<0.001
Cephalosporin resistance	1 (1.7%)	0 (0%)	1 (0.2%)	10 (0.9%)	0.303
Erythromycin resistance	25 (40.3%)	15 (16.0%)	81 (18.6%)	236 (21.6%)	<0.001
Levofloxacin resistance	0 (0%)	1 (1.1%)	4 (0.9%)	25 (2.4%)	0.181
Multiresistance	12 (20.3%)	3 (3.3%)	14 (3.3%)	74 (6.9%)	<0.001

**Table 2:** Frequency (*n* and %) of antimicrobial resistance among the 3 most frequent serotypes (3, 8, and 12F) and other serotypes (Other).

Serotype	3 (*n* = 299)	8 (*n* = 314)	12F (*n* = 149)	Other (*n* = 1071)	Overall (*n* = 2076)
Resistant to:					
Penicillin G	7 (3.0%)	4 (1.6%)	1 (0.8%)	114 (13.1%)	148 (8.8%)
Cephalosporin	0 (0%)	1 (0.4%)	0 (0%)	9 (1.0%)	12 (0.7%)
Erythromycin	42 (18.0%)	10 (4.0%)	4 (3.2%)	258 (29.2%)	357 (21.2%)
Levofloxacin	4 (1.7%)	0 (0%)	1 (0.8%)	18 (2.1%)	30 (1.8%)
Multiresistant	4 (1.7%)	2 (0.8%)	0 (0%)	81 (9.6%)	103 (6.3%)

**Table 3:** Results from logistic regressions evaluating the association between 2010 vaccination policy and antimicrobial resistance (to penicillin G, to erythromycin, and multiresistance). Odds ratios (ORs), 95% confidence intervals (95%CIs), and *p*-values are provided.

	Penicillin G Resistance			Erythromycin Resistance			Multiresistance		
Predictors	ORs	95%CI	*p*-Value	ORs	95%CI	*p*-Value	ORs	95%CI	*p*-Value
2010 vaccination policy: After	3.87	1.29–12.8	0.019	0.86	0.36–2.1	0.734	3.78	1.00–16.9	0.060
Age category (ref. Child): Infant	3.64	1.37–10.4	0.012	5.08	2.27–12.0	<0.001	11.5	3.15–56.9	0.001
Serotype included in the PCV 13 vaccine: Yes	5.98	1.98–20.3	0.002	4.30	1.70–11.8	0.003	10.6	2.61–56.7	0.002
Observations	157			156			150	150	150
R^2^ Tjur	0.134			0.177			0.227	0.227	0.227

## Data Availability

The original contributions presented in the study are included in the article, further inquiries can be directed to the corresponding author.

## Supplementary Materials

The following supporting information can be downloaded at: https://www.mdpi.com/article/10.3390/antibiotics13080740/s1, Table S1: Overall sample characteristics; Table S2: *S. pneumoniae* serotypes; Figure S1: Annual relative frequencies of antibiotic resistance stratified by age group.
