# Supplementary material for: Epidemiology and Impact of Anti-Pneumococcal Vaccination and COVID-19 on Resistance of Streptococcus pneumoniae Causing Invasive Disease in Piedmont, Italy
Source: Antibiotics (Basel). 2024 Aug 6;13(8):740. doi: 10.3390/antibiotics13080740 (PMC11350834; doi:10.3390/antibiotics13080740)
Supplement: Supplementary file 1 [file antibiotics-13-00740-s001.zip › antibiotics-3101726-supplementary.pdf]

Table S1. Overall sample characteristics. Median [IQR] is reported for quantitative variables, while absolute and relative (%) frequencies for categorical variables.

	**Overall (*n* = 2076)**	**Missing *n* (%)**
Year of collection		-
2008	27 (1.3%)	
2009	20 (1.0%)	
2010	23 (1.1%)	
2011	13 (0.6%)	
2012	79 (3.8%)	
2013	172 (8.3%)	
2014	156 (7.5%)	
2015	193 (9.3%)	
2016	202 (9.7%)	
2017	316 (15.2%)	
2018	236 (11.4%)	
2019	243 (11.7%)	
2020	97 (4.7%)	
2021	81 (3.9%)	
2022	143 (6.9%)	
2023	75 (3.6%)	
Age, years	70.1 [55.8-80.1]	7 (0.3%)
Age category		-
Infant	74 (3.6%)	
Child	118 (5.7%)	
Adult	550 (26.5%)	
Elderly	1334 (64.3%)	
Sampled material		11 (0.5%)
Blood	1812 (87.7%)	
Cerebrospinal fluid	253 (12.3%)	
Sepsis	913 (44.0%)	-
Meningitis	264 (12.7%)	-
Serotype included in the PCV13 vaccine	760 (36.6%)	-
Serotype included in the PCV10 vaccine	354 (17.1%)	-
Most frequent serotypes		-
3	298 (14.4%)	
8	314 (15.1%)	
14	98 (4.7%)	
Other	1366 (65.8%)	
Penicillin G antibiogram		385 (18.5%)
Susceptible	1543 (74.3%)	
Intermediate	80 (3.9%)	
Resistant	68 (3.3%)	
Penicillin G resistance	148 (8.8%)	385 (18.5%)
Cephalosporin antibiogram		388 (18.7%)

	Overall<br>(*n* = 2076)	Missing<br>*n* (%)
Susceptible	1676 (80.7%)	
Intermediate	50 (2.4%)	
Resistant	12 (0.6%)	
Cephalosporin resistance	12 (0.7%)	388 (18.7%)
Erythromycin antibiogram		393 (18.9%)
Susceptible	1326 (63.9%)	
Intermediate	7 (0.3%)	
Resistant	357 (17.2%)	
Erythromycin resistance	357 (21.2%)	393 (18.9%)
Levofloxacin antibiogram		443 (21.3%)
Susceptible	1374 (66.2%)	
Intermediate	229 (11.0%)	
Resistant	30 (1.4%)	
Levofloxacin resistance	30 (1.8%)	443 (21.3%)
Multiresistance	103 (6.3%)	433 (20.9%)

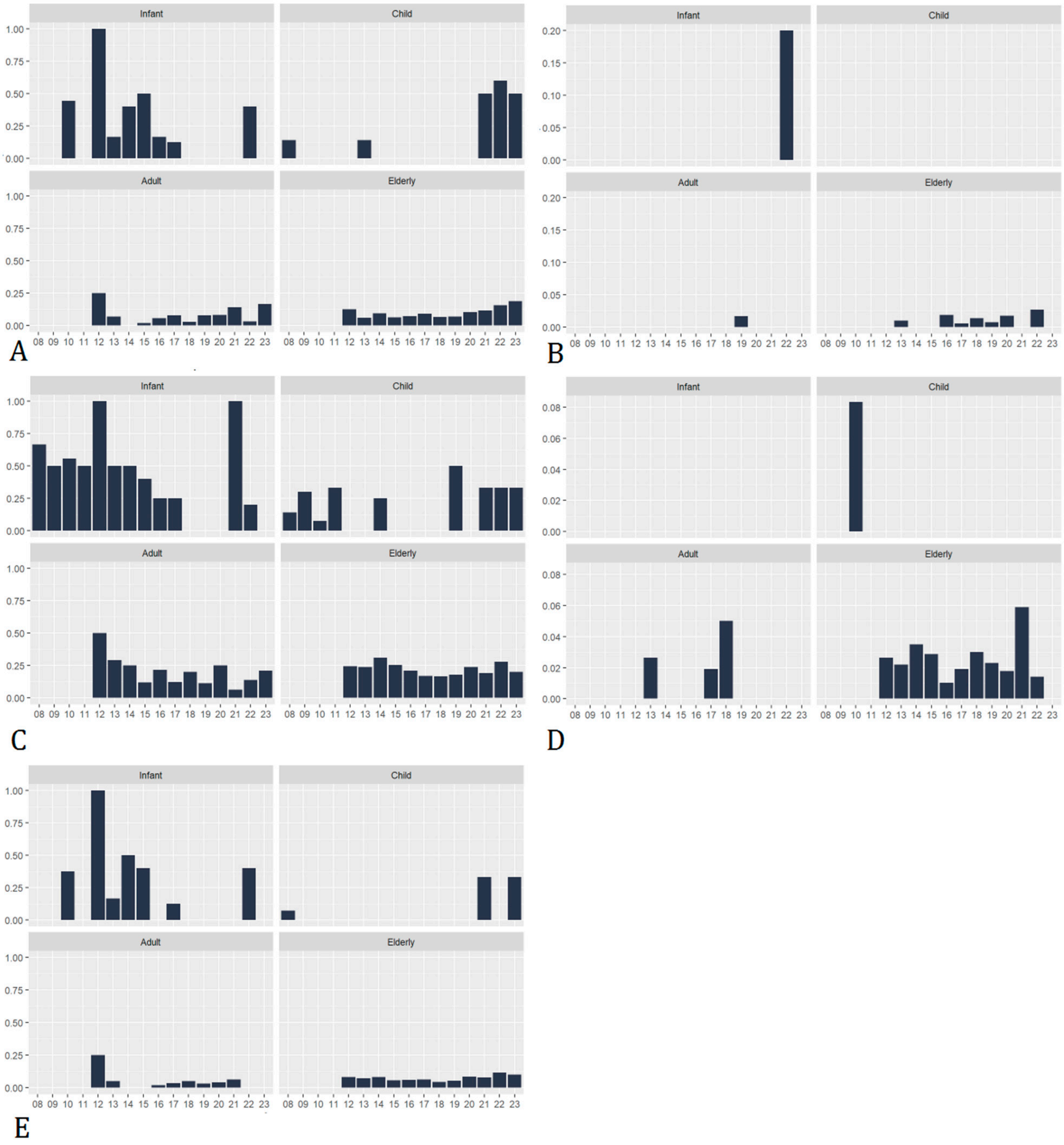

Figure S1. Annual relative frequencies of antibiotic resistance stratified by age group; A: penicillin; B: cephalosporin; C: erythromycin; D: levofloxacin; E: multi-resistance

Table S2. *S. pneumoniae* serotypes. Absolute frequencies for each serotype in the study sample are reported.

Serotype	*n*
**8**	314
**3**	299
**12F**	149
**14**	98
**22F**	76
**19A**	65
**1**	61
**7F**	57
**10A**	47
**6A**	42
**18A**	41
**4**	40
**9N**	38
**23A**	36
**11A**	35
**23B**	35
**19F**	33
**20**	33
**15A**	28
**15B**	27
**6C**	22
**33F**	20
**9L**	20
**9V**	19
**29**	17

Serotype	*n*
**23F**	16
**6B**	15
**38**	14
**15C**	12
**31**	12
**34**	12
**2**	11
**5**	11
**24F**	7
**7A**	6
**12B**	5
**18F**	5
**9A**	5
**12A**	4
**18C**	4
**33A**	4
**33B**	4
**35F**	4
**22A**	3
**33D**	3
**16**	2
**18B**	2
**24A**	2
**35B**	2
**9B**	2

Serotype	*n*
**10F**	1
**10C**	1
**11C**	1
**11F**	1
**23**	1
**24B**	1
**9**	1
**Not typed**	250
